# Gamification in Stress Management Apps: A Critical App Review

**DOI:** 10.2196/games.7216

**Published:** 2017-06-07

**Authors:** Alexandra Hoffmann, Corinna A Christmann, Gabriele Bleser

**Affiliations:** ^1^ Junior research group wearHEALTH Department of Computer Science University of Kaiserslautern Kaiserslautern Germany

**Keywords:** game element, mHealth, motivation, app, behavior change, gamification

## Abstract

**Background:**

In today’s society, stress is more and more often a cause of disease. This makes stress management an important target of behavior change programs. Gamification has been suggested as one way to support health behavior change. However, it remains unclear to which extend available gamification techniques are integrated in stress management apps, and if their occurrence is linked to the use of elements from behavior change theory.

**Objective:**

The aim of this study was to investigate the use of gamification techniques in stress management apps and the cooccurrence of these techniques with evidence-based stress management methods and behavior change techniques.

**Methods:**

A total of 62 stress management apps from the Google Play Store were reviewed on their inclusion of 17 gamification techniques, 15 stress management methods, and 26 behavior change techniques. For this purpose, an extended taxonomy of gamification techniques was constructed and applied by 2 trained, independent raters.

**Results:**

Interrater-reliability was high, with agreement coefficient (AC)=.97. Results show an average of 0.5 gamification techniques for the tested apps and reveal no correlations between the use of gamification techniques and behavior change techniques (*r*=.17, *P*=.20), or stress management methods (*r*=.14, *P*=.26).

**Conclusions:**

This leads to the conclusion that designers of stress management apps do not use gamification techniques to influence the user’s behaviors and reactions. Moreover, app designers do not exploit the potential of combining gamification techniques with behavior change theory.

## Introduction

In today’s society, many people suffer from chronic exposure to stress [1], which is known to be related to mental as well as physical health problems (eg, depression, cardiovascular, and gastrointestinal diseases) [2]. In fact, the American Psychological Association reported both an increase in health problems caused by stress [3] and the experience of stress symptoms in three quarters of the American population [4].

A person’s well-being, however, does not solely depend on his or her exposure to stress, but also on the way he or she copes with stress [5]. Coping techniques aim at the reduction, tolerance, or elimination of stress and stress triggers [6]. These techniques are normally taught in single therapy or group interventions. In comparison with this conventional treatment, smartphone apps designed for stress management have been suggested to facilitate considerable financial savings in health care [7]. They also allow users to complete the training in a time and place convenient to them [8]. This idea follows the recent mobile health (mHealth) trend. It aims to help people improve their health through mobile technologies [9] by affecting the user’s education, motivation, and adherence [10,11]. mHealth is already being applied to support mental as well as physical health programs [12] and is met with broad acceptance [13].

Stress management apps, like all behavior change programs, must be based on evidence-based content from behavior change theory, such as behavior change techniques [14] and stress management methods [15] to ensure effectiveness [9,16,17] through long-term behavior change [18]. While important, the use of evidence-based content alone has been considered as insufficient to ensure adequate user engagement and motivation [19]. However, both of these aspects have a great influence on an individual’s exposure to an intervention program [20]. Since exposure rates are directly linked to effectiveness [19], the integration of gamification techniques in order to increase motivation and engagement in behavior change contexts is an important research topic.

Gamification, that is, the use of game elements in nongame contexts, is aimed at making interventions (including mobile apps for behavior change) more enjoyable, motivating, and engaging [21]. As a result, this approach could pose a possible solution to the lack of motivation to follow self-management procedures and to care for oneself, which are often observed in health-related contexts and chronic illnesses [22]. In view of these possibilities, gamification has been suggested to positively influence user behavior and lifestyle [23]. Moreover, it offers a way to provide the user with a feeling of empowerment regarding health-related content and can create a new type of interaction between the user and the app content [24].

Indeed, gamification has already been suggested to positively influence user self-management [22,25]. Moreover, it proved to have positive effects on health and behavioral outcomes [24] and the retention of desired user behaviors [26]. These positive effects are most likely the result of comprehensive motivational support and invoking flow experiences [27,28]. Gamification also helps to make the user feel represented and in control [22] by adjusting techniques to the user’s motives [27] and abilities [29]. Invoking user motivation through gamification in this manner is an important way to keep the user’s interest [30] and, thus, to increase his or her exposure to the evidence-based content [18].

In fact, it could already be shown that the implementation of gamification in form of rewards for diabetes patients [31] and combinations of gamification techniques for weight management in children can be effective in promoting behavior change through apps [32]. This is further supported by Hamari [33], who showed that the use of gamification techniques can, indeed, increase the use of a service.

Regardless of these facts, gamification in the context of health and wellness [34] as well as the use of gamification aspects in apps targeting health behavior change has only been rarely investigated so far. Mendiola et al [35] investigated the use of gamification (defined as the use of badges, points, and levels) in 234 health apps and found that only 11.5% of the reviewed apps made use of gamification. In contrast to this finding, a study by Schoffman et al [36] classified 57 apps aimed at pediatric weight loss, healthy eating, and physical activity with regard to being a game. They found that 56% of the apps included in their sample matched their criteria for a game. A third study by Payne and colleagues [37] reviewed 52 physical activity game apps with respect to 10 gamification aspects and found that all of the reviewed apps included at least one gamification technique. Moreover, their study found no correlation between the investigated gamification techniques and health behavior theory constructs [37]. In a fourth study, Lister and colleagues [38] reviewed 132 apps from the Apple iTunes Store aimed at health and fitness with regards to their inclusion of 13 behavior change techniques, 6 gamification techniques, and 10 game elements. They revealed a correlation of gamification techniques with both game elements and the evidence-based content, whereas the use of game elements was also correlated with app popularity.

Interestingly, the association between gamification techniques found by Lister and colleagues was only due to the motivational behavior change aspects, namely, social support, providing incentive, goal setting, cognitive strategies, and self-efficacy. Regarding the use of theoretical content, the authors concluded that these apps were greatly lacking in all three categories (behavior change techniques, gamification techniques, and game elements). These findings are in accordance with the assertion that the development of health apps is currently lacking efficient and selective implementation of gamification [39]. Whereas the same might be assumed with regards to stress management apps, the implementation of gamification techniques in the context of stress management has only been studied with respect to the distinction between extrinsically and intrinsically motivating aspects [40]. However, the use of specific gamification techniques has so far never been studied in the context of stress management.

According to the mechanics, dynamics, and aesthetics (MDA) model of Hunicke at al [41], three levels of a gamified experience can be distinguished. The first level, mechanics, refers to the implementation of gamification techniques. As such, this level is immediately visible to the user and can be directly influenced by the designers of an app. Moreover, this level of gamification implementation has great impact on the user’s behavior and reactions [41]. The second and third stages are dynamics and aesthetics. In contrast to the first level, these levels can only be influenced by app designers in an indirect way. Both, dynamics and aesthetics, refer to the effects that the use of gamification techniques has on the user [41]. Whereas it is important to determine the effects and reactions a gamified experience causes, it first needs to be investigated whether app designers even make use of gamification techniques. For this reason, an expert review of apps available in the Google Play Store was conducted in order to investigate whether app designers try to influence user behavior through the integration of gamification in the context of stress management.

For this purpose, an extended taxonomy of gamification techniques was developed. As no universal list of game elements exists, a list of features that are found in most but not necessarily in all games [42] was collected. In a first step, this list was based on a publication by Lister and colleagues [38], who identified a total of 6 gamification techniques. Their study distinguished between game elements and gamification. Based on the definition of gamification as the use of game design elements in nongame contexts [42], this study did not differentiate between gamification techniques and game elements. As a result, the taxonomy was further extended. In the second step, a literature search for a list of elements that are characteristic to games was conducted. This search was based on the more general search terms gamification, gamification techniques, and game elements. It resulted in a list of common gamification techniques by Reeves and Read [43], which were added to the taxonomy. In the next step, two more items, “agent” and “secondary game objectives” that were found during the literature research were added. “Agents” have been used in health [44,45], learning-related [46], and behavior change [47-49] contexts for some time now. “Secondary game objectives” have been described as a fundamental element of game design [50]. Examples for the application of this gamification technique include, “World of Warcraft’s” crafting system, “Cut the Rope’s” option for star collection, and “Pirates!” choice to challenge other captains [51]. After establishing a list of common gamification techniques, a literature search was conducted with the specific names of the identified gamification techniques. The purpose of this specific search was to provide item definitions that are common and easy to understand. This strategy resulted in a taxonomy of 17 gamification techniques and their accompanying definitions (see Table 1).

In the last step, all 17 gamification techniques were assigned to one of four categories: economic, social, performance-oriented, or embedding-focused. Economic gamification techniques include economical aspects such as “rewards” [52], which are frequently used in interventions [53], and “economies” [43], that mirror those of the real world. Social gamification techniques have a primary focus on social aspects and, thus, provide social interaction for the user with virtual characters as well as techniques that supply the opportunity for social interaction with other users. Examples for social gamification techniques include “avatars” and “teams” [43]. Performance-oriented gamification techniques such as “leaderboards” [42] and “feedback” [43] offer information on the user’s performance, either in comparison to his or her own previous performance or to the performance of other users, or without direct comparison. Embedding-focused gamification techniques are aimed at the environmental setting and include “three-dimensional (3-D) environments” and “narrative context” [43]. The consequent coding manual in Table 1 provides all investigated gamification techniques ordered by category as well as exact definitions to ensure interrater-reliability.

In addition to the number of used gamification techniques, this study also examined the correlation between gamification and the evidence-based content as presented by Christmann and colleagues [54]. They investigated the use of effective behavior change techniques based on a taxonomy provided by Abraham and Michie [14] and emotion-focused stress management methods in the same body of apps as this study.

This study was the first to investigate the use of gamification techniques in apps aimed at stress management. Its goal was to provide important information on whether designers of stress management apps are trying to influence user behavior through the use of gamification.

## Methods

### Study Design

This study investigated the use of gamification techniques in stress management apps available through Google Play. The selected apps were reviewed by 2 trained, independent raters on their inclusion of 17 gamification techniques (see Table 1 in the Methods subsection Evaluation). Further, the apps were reviewed in regard to a number of additional, relevant aspects (eg, connection to social networks, inclusion of advertisement). Their detailed definitions are provided in Table 2 in the Methods subsection Evaluation.

### App Selection

This review included free apps that were available through Google Play. Apps were identified by using the search terms “stress management,” “stress reduction,” and “stress relief.” For each search term, the first 250 apps were examined according to the following inclusion and exclusion criteria.

First, duplicates and apps not found in the categories “health and fitness” or “ medical” were eliminated. With 563 apps being excluded at this stage, 187 apps remained. Their descriptions were reviewed with the constraint that they had to be in English and aimed at stress management, health, or wellness for healthy adults. Thus, apps whose descriptions focused on children, adolescents, and older adults (n=5) were excluded. In addition, apps targeting anxiety, depression, diabetes, insomnia as well as other medical conditions (n=82), addiction (n=2), weight management (n=13), or beauty and cosmetics (n=2) were excluded from this study. The same was done with apps that clearly focused on content other than stress management (n=8) and apps that could only be used with a wearable device (n=2). Therefore, 73 apps were downloaded and assessed for eligibility.

**Table 1:** Taxonomy of 17 gamification techniques.

Technique		Definition
**Economic**		
	Marketplace and economies [43]	Offering a virtual currency that establishes an economy in which the user may trade, purchase, auction, receive a salary, and so on as he or she would in real life economy.
	Digital rewards [38,42,52,57,58]	Include, for example, badges (signal status, aesthetic value), game currency, points, and resources or property (experience points, health, houses); virtual goods (objects, food), powers or abilities (increase as the player progresses), add to record of achievements and validation (marks of approval from others).
	Real world prizes [38,52]	Include, for example, deals or discounts (similar to a loyalty program), financial prizes (cash prize, voucher), goods or services (tote bag, free massage, car, parking spaces, health savings account contributions, insurance contributions), time (time saved, vacation or time off), and lottery or draw or bet for any of the above.
**Social**		
	Avatar [43]	Ability to represent oneself through a virtual character within the media and excerpt precise control over that representation.
	Agent [45,46,59,60]	A virtual character that does not represent oneself and provides instructions or support (eg, social support).
	Competition [38,43]	Competition with other players or between teams to achieve new levels, ranks, reputations through winning challenges, selling digital rewards, building spaces, creating materials, and so on, that are restricted by rules, which are either provided by the program, or user-generated and apply to everyone.
	Teams [43,52]	Program involves multiple players, who interact and form relationships that allow for collaborative achievements (eg, guilds, multiplayer modes).
	Parallel communication systems [43]	Allow for interaction with other players via different channels (eg, private, public) through headsets, text, email, and so on within the application.
	Social pressure [38,52,57,61]	Competitions within or between teams that give the user the feeling he or she has to take part in events (eg, a quest) in order to avoid social consequences. The user is pressured to perform in order to be invited to a further raid or quest or event; feels he or she is needed and, therefore, does not want to let other users down.
**Performance-oriented**		
	Feedback [14,43]	Text or spoken language, visual or auditory feedback that is either temporary or constant and evaluates the user’s performance in relation to a set standard or other’s performance.
	Levels [38,43,62]	Levels provide information on the stage of the game. Usually a specific number of points or experience is required in order to reach the next level. New levels can be shown through, for example, differences in optical design, rise in rewards, and increase in difficulty.
	Secondary game objectives [42,50,51]	Optional aspects or layers or challenges or secondary goals of play (find as many treasures vs complete as soon as possible) that reward the player upon completion or simply exist for their own sake.
	Ranks of achievement [43,52,62]	Measurement of character development with regards to position and value of a player or player’s avatar in the program community.
	Leaderboards [38,42,57,58,62]	The purpose of a leaderboard is to make simple comparisons by displaying players at the same or different levels, ranked by proximity and recency to oneself. They can be based on player feedback, scores, and promotion.
	Time pressure [42,43]	Time limits set for completion of tasks or the duration of the usability of specific skills, occurrences, and objects (excluding countdowns on videos and audios).
**Embedding-focused**		
	Narrative context [43,58]	Back stories that guide the action and help to organize character roles, rewards, and group action.
	3-D^a^ environments [43]	Rendering 3-D graphical models of physical properties that parallel those in the real world, on a 2-dimensional screen.

^a^3-D: three-dimensional.

**Table 2:** Taxonomy of 8 additional aspects.

Item	Definition
Connection to social network	The app itself provides a connection to a social network (eg, Facebook, Twitter)
Advertisement	Pop-up or stationary advertisements are shown within the app
Registration or account	Registration is required in order to use the app or some functions of the app
Pure e-book^a^	The app consists only of text that may or may not be divided into different chapters
Test version	Payment or download of a full version is necessary to receive access to some features of the app
Internet connection necessary	The app only opens when an Internet connection is available
External links to other websites	Websites are linked in the app, or videos or audios only play with an Internet connection
Wearable	The app can also be used with a wearable device

^a^e-book: electronic book.

**Figure 1:**
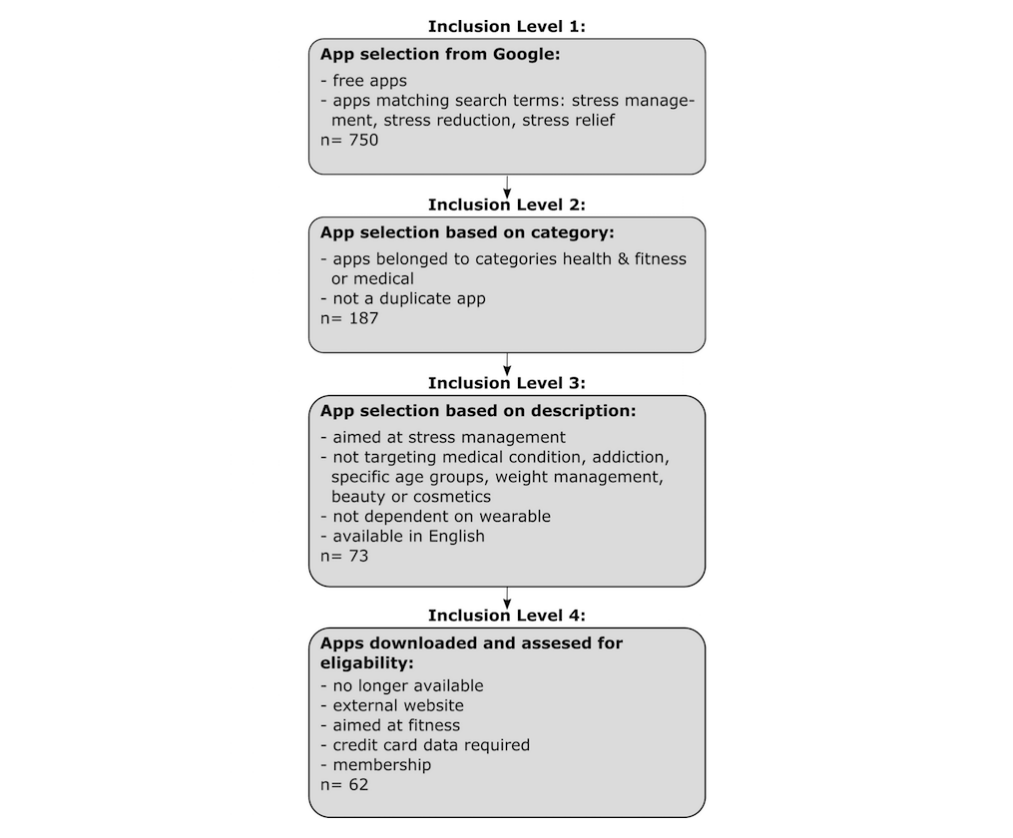
Levels and criteria for inclusion of identified apps.

Eleven additional apps had to be excluded during the review process. Since the apps were reviewed over a total of 1 month, 3 of the initially selected apps were no longer available at the time of testing. One app had to be excluded during the review process because it could only be used after entering the user’s credit card data, whereas another app turned out to be solely focused on fitness aspects without any further indication toward stress reduction. One app consisted only of an external website. Another 4 apps turned out to be only accessible via a membership or company code, whereas yet another app could only be used with a wearable device. A basic outline of the selection process can be found in Figure 1. A more detailed diagram of the app selection process can be found in Christmann et al [54].

Apps that met all inclusion criteria (N=62) were downloaded, installed, and tested by 2 trained, independent raters in October 2015. For this, raters used the device emulator of the development environment Android Studio 1.3 running Android OS 4.4 Android Studio [55]. This approach was not always successful regarding the presentation of some app contents, such as, playing of audio or video, download of data, and display of pages. Therefore, apps for which such problems occurred were subsequently installed on a Nexus S Android smartphone, where both reviewers examined the problematic features.

### Evaluation

As is common procedure [36,56], the apps in this study were reviewed by 2 previously trained, independent raters according to the taxonomy of gamification techniques (Table 1). Reviewer 1 had a background in psychology and reviewer 2 had a background in cognitive science. Both reviewers studied the taxonomy in detail and practiced the evaluation process on approximately 30 apps that had previously been excluded from the study during the selection process. Unclear item descriptions were discussed and revised during this testing phase. This process aimed to ensure a comprehensible and applicable taxonomy. After the training phase was completed, the reviewers went on to review the apps that had met the selection criteria independently from each other.

**Figure 2:**
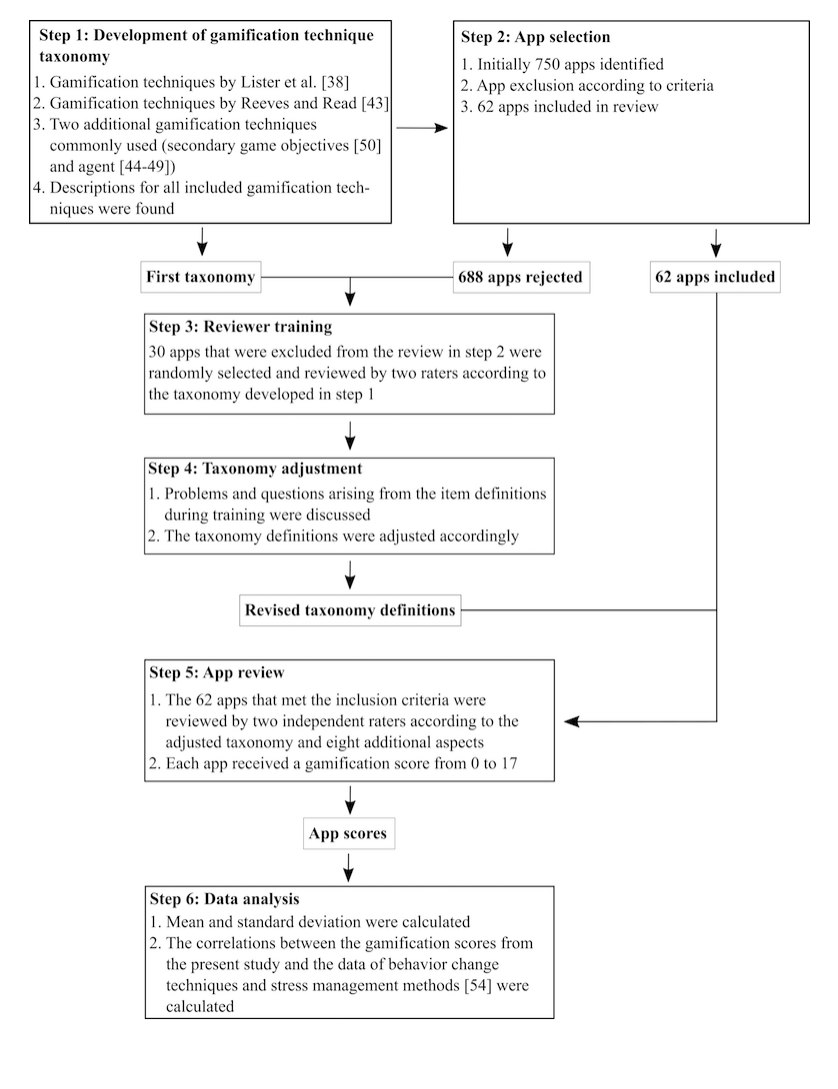
Outline of the overall study procedure.

The reviewing process of this study only included content that was provided by the app itself. Information and features on websites that were linked within the app were not considered. Because all apps allowed the user to progress at his or her own speed, both raters could thoroughly check all features of the apps until it was apparent that no new content was going to be activated. An overall outline of the study procedure is illustrated in Figure 2.

Each app received a score between 0 and 17, representing the number of gamification techniques included in the app. If the raters disagreed on the use of a technique, it was noted as included. Supplementary, it was noted whether an app needed an Internet connection to run, showed advertisements, required registration, had additional features available for payment, used links to external websites, and could be used with wearables (Table 2).

### Analysis

To make sure that the evaluation criteria were applied in a consistent manner by both raters, the interrater reliability was calculated according to Gwet’s agreement coefficient (AC) [63]. This study applied Gwet’s AC instead of the more often utilized Cohen kappa, because Cohen kappa is only reliable if the trait prevalence is approximately 50% [63,64]. However, this is not the case for this study (the prevalence is very low), making Gwet’s AC more reliable in representing the gathered data. Results show a high interrater agreement of AC=.97.

Mean, standard deviation, and range were calculated for the sum of gamification techniques. To determine whether a linear relationship exists between the occurrence of the content from behavior theory and gamification techniques, correlational analyses were performed. For this, the Spearman correlation coefficient r and statistical significance *P* was calculated for the number of gamification techniques and the scores of behavior change techniques as well as for stress management methods in each app [54].

## Results

### Evaluation of Gamification Techniques

The 62 stress management apps included an average of 0.5 (range 0-2) gamification techniques, with a standard deviation of 0.7. The sum score of gamification techniques for each app (presented in Multimedia Appendix 1) reveals that 8 out of 62 apps included a total of 2 techniques, and 12 apps included 1 technique, whereas 42 of the reviewed apps did not include any gamification techniques.

**Figure 3:**
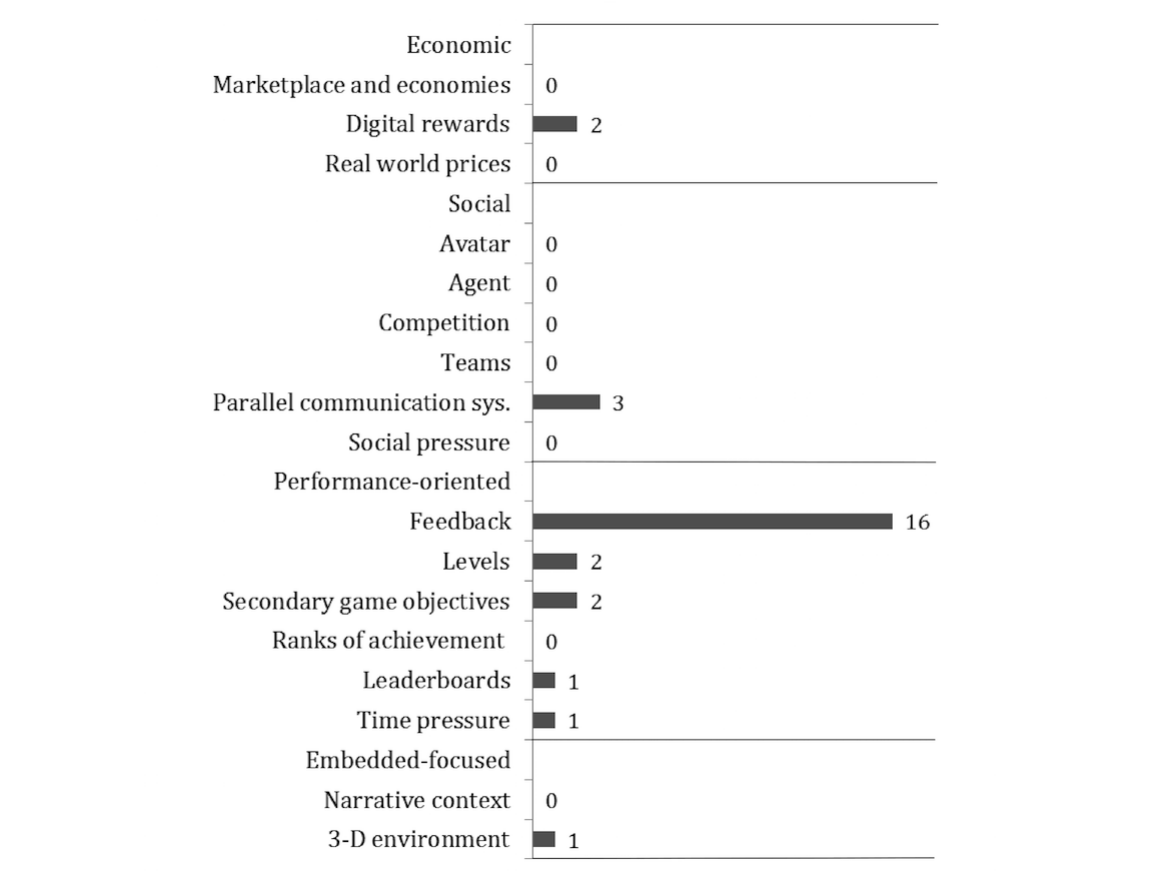
Frequencies of the 17 gamification techniques included in the apps. Techniques were scored according to the previously introduced taxonomy of gamification techniques (see Methods subsection Evaluation).

Regarding frequency of use, “feedback” (n=16) was implemented most often, followed by “parallel communication systems” (n=3). In contrast, the aspects “social pressure,” “real world prizes,” “teams,” “competition,” “marketplace and economies,” “ranks of achievement,” “narrative context,” “agents,” and “avatars” were never included (Figure 3). To summarize, the performance-oriented gamification techniques were found most frequently.

### Evaluation of Additional Aspects

**Figure 4:**
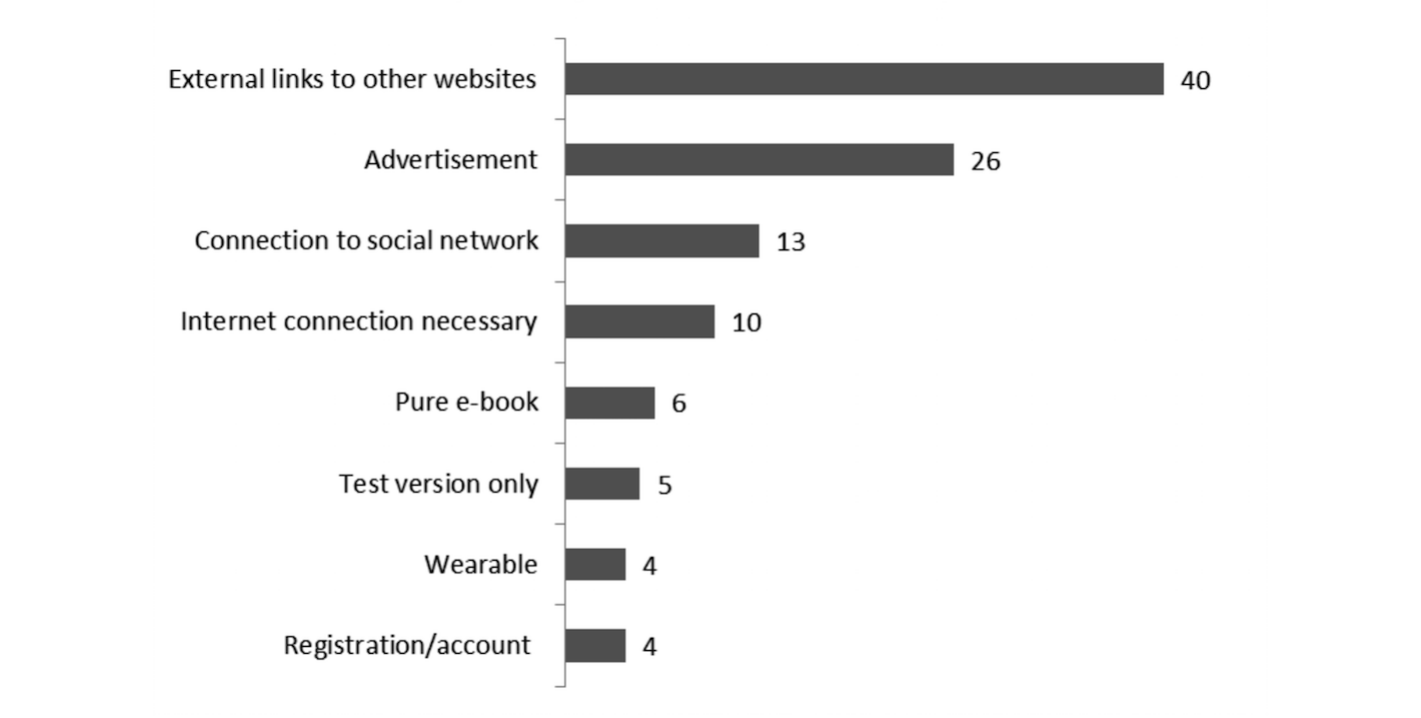
Frequencies of 8 additional aspects included in apps. Additional aspects were scored according to the previously introduced taxonomy for additional aspects (see Methods subsection Evaluation) and are ranked by the most frequently ones included.

Beside the use of gamification techniques, this study also considered several additional aspects regarding the selected apps. In this context, it has to be emphasized that a considerable number of apps included external links to other websites (n=40), thus utilizing additional sources for features and information. The display of advertisements was found in as many as 26 apps. Thirteen of all reviewed apps required Internet connection. Furthermore, 6 apps only included text content and were, therefore, rated as pure electronic book (e-book). The frequencies of all additional aspects investigated in this review are displayed in Figure 4.

### Correlation Analysis

Since the gamification data was positively skewed (*P* ≤.01), the Spearman correlation was applied to see whether an association existed between the use of gamification techniques and the use of the evidence-based content [54]. “Feedback” was excluded from this analysis as it is a gamification technique as well as a behavior change technique and was included in both taxonomies. The results for the correlation analysis revealed that no significant associations between gamification techniques and behavior change techniques (*r*=.17, *P*=.20) as well as gamification techniques and stress management methods (*r*=.14, *P*=.26) could be found.

## Discussion

### Principal Findings

The goal of this study was to investigate the use of gamification techniques in apps that are aimed at stress management for healthy adults, available for free through Google Play. By conducting an expert review to investigate the use of gamification techniques in a quantitative manner, this study focused on the first level of the MDA framework [41]. The purpose of this approach was to find out whether designers of stress management apps are currently making use of gamification techniques in order to influence the user’s behavior. The evaluation was based on a taxonomy of 17 gamification techniques, which expands the range used in previous reviews (eg, [37,38]). This extended taxonomy combines the gamification techniques used in an app review by Lister and Colleagues [38] and a more extensive list of game elements developed by Reeves and Read [43]. Moreover, the present taxonomy provides clear item definitions for the investigated gamification techniques to ensure interrater-reliability (AC=.97).

Results showed an average as low as 0.5 gamification techniques for the 62 tested apps. Although at least one technique was included in 32% (20/62) of the apps, no app included more than 2 techniques. In fact, as much as 68% (42/62) of the sample did not use any gamification technique at all. Thus, even though some app developers tried making use of gamification to some extent, these findings indicate little to no use of gamification techniques in the context of free stress management apps for Android. Therefore, it can be concluded that app designers have not been trying to impact user behavior through the implementation of gamification techniques.

These results contradict those presented by Payne et al [37], who found that their sample (52 physical activity game apps from the Apple Store) included all of the 10 investigated gamification techniques once, except “real world prizes.” This high utilization of gamification techniques compared with the results of this study could be explained by the fact that their review focused on game apps. In a similar study, Lister and colleagues [38] investigated the use of 6 gamification techniques in 132 fitness and health apps from the Apple Store. Whereas their gamification scores were slightly larger than in this study, Lister and colleagues’ conclusion confirms the implications of this review, namely, that there is a lack of use for gamification techniques.

Regarding frequency of use, “feedback” was the most often implemented technique. This is a positive result, as “feedback” is not only a gamification technique but also a common technique for promoting behavior change [14]. It has the potential to increase the effectiveness of an intervention, for example, by offering important information, providing social comparison, and helping the user to make decisions [65]. The second most often used technique proved to be “parallel communication systems.” This technique can among others, be used in combination with teams. Both “parallel communication systems” and “teams” pose social aspects and can, therefore, provide an important means for social support [22].

These findings differ significantly from those of Lister and colleagues [38], who reported “social peer pressure,” “digital rewards,” and “competition or challenges” to be the gamification techniques most often used. These strong variations are most likely due to the difference in app genre.

Whereas some apps reviewed in this study made use of “digital rewards,” “levels,” and “secondary game objectives,” this was only the case to a very small degree. These aspects were only found in 2 apps. Nonetheless, app designers should make more intensive use of these techniques. As rewards are a common feature in other gamified interventions, designers could provide points [53], a token system, or badges to ensure a more active use of the system [66]. “Secondary game objectives” should also be considered for implementation more often, although, designers should bear in mind that this technique is only effective when these objectives support the primary goal of the app [50]. “Levels” can be used to provide goals [62] and show progression but at the same time, they can promote competition, which might not be sought in the context of stress management.

The gamification techniques “3-D environments,” “leaderboards,” and “time pressure” were included only once. On the one hand, game designers should consider to make stronger use of “3-D environments,” as such environments may elicit an enhanced recovery from stress [67]. On the other hand, care should be taken when implementing “time pressure” and “leaderboards.” Whereas the use of these techniques surely makes sense in other contexts, for example, exercising, it might lead to excitement and tension in the user. As a result, the use of these 2 gamification techniques in stress management apps might counteract the overall aim of relaxation. Since the argument of causing stress for a user is also valid for the items “competition” between users and “social pressure,” it is favorable for the tested apps that these aspects were not detected by the investigators.

Other techniques that were never used included “marketplace and economies,” “real world prizes,” “narrative,” “avatar,” “agent,” “teams,” and “ranks of achievement,” App designers should consider a more extensive use of these techniques. As such, the implementation of a “marketplace and economy” (eg, through a currency) can help to quantify the value of rewards and objects [43]. It could also be combined with “real world prizes,” which could, for example, be implemented through a loyalty program. Even though “real world prizes” are especially useful to win over new users, designers should keep in mind that excessive use of this technique can habituate players [52]. The implementation of a “narrative” provides the user with information on what to do and thus helps to achieve goals [43], such as the change of a behavior. The use of an “avatar” personalizes the experience for the player and indicates his or her role in the narrative. This technique is most effective if the avatar resembles the person with whom it is interacting [43]. Moreover, the creation of an “agent” to represent another person can help the user to accomplish different goals and tasks. It can also have positive effects on their learning. A reason for this might be the ability of an “agent” to explain [46] and, thus, to guide the user through actions or words. These aspects can increase the general interactivity of apps and can cause the perception of social interaction. Another way to incite social interaction and provide interactivity is the use of “teams.” “Teams” cause a social relationship to form between users [43]. However, the use of “teams” could also cause social pressure, which designers may want to avoid in this context. The same might be true for “ranks of achievement” as these are often visible to other users and can be used to express a user’s status in relation to others. Whereas it might lessen the effectiveness of this technique, a way to avoid such a negative outcome could be to keep the rank invisible to other users [43].

The techniques reported to be used least often in this study differ from those by Lister et al [38] and Payne et al [37]. In contrast to the results of this study, Lister and colleagues found that 25% of their sample used “real world prizes,” 33% “leaderboards,” and 25% “ranks of achievement,” whereas Payne et al [37] reported 19% for the use of “rankings or standings” and 29% for “leaderboards.”

Considering that neither the use of evidence-based content, nor gamification techniques alone is sufficient to ensure both behavior change and app usage [19,20], the lack of gamification techniques discovered in the reviewed sample appears questionable. Furthermore, no association between gamification techniques and behavior change techniques, or between gamification techniques and stress management methods could be detected. This reveals that the reviewed apps did not use combinations of gamification and evidence-based content.

The lack of correlation found in this study does not match the results of Lister and colleagues [38], who reported a correlation between gamification techniques and specific motivational behavior change techniques. This disagreement between Lister et al and this study might be due to the difference in app genre targeted by the 2 studies. In addition, Lister and colleagues reviewed twice as many apps as compared with this study, which allows uncovering relations with smaller effect sizes. Opposed to this, Payne et al [37] reported that no correlation between gamification techniques and behavior change techniques existed for their sample. Their results support the findings of this study that app developers should pay more in-depth attention to the use of gamification techniques and their sensible combination with evidence-based content in apps aiming at behavior change.

Because the implementation of gamification techniques is directly influenced by app designers and can largely affect user behavior and reactions, designers should carefully consider the effects specific gamification techniques might have on the user. Correspondingly, designers should chose techniques with strong regard to the context of the app they are constructing. Hence, future studies should pay close attention to the levels of dynamics and aesthetics [41] and, thereby, to the functions and effects the applied gamification techniques have on the user’s behavior and reactions.

The need for improvement suggested by the gamification results also extends to the additional aspects that were investigated in this study. As such, a large portion of the apps included features that require an Internet connection. This approach reduces the time that is needed for installation as well as download and provides the opportunity for larger content. Nevertheless, this aspect might require optimization, since its use makes apps dependent on Internet connections, which may not be available at all times and in all places. This point is even more important for the 10 apps that ran only when an Internet connection was available. Another aspect that needs to be addressed in this context is the fact that as many as 6 out of 62 apps consisted of text only. Therefore, it is hard to see the advantage of such apps over self-support e-books and websites—consumers expect modern technology to be interactive. The user’s perception of the media’s interactivity has great influence on user loyalty [68]. As such, instead of text only, these apps could make use of social and community aspects [22], or react to the user in order to create flow experiences [29]. However, most apps did not make use of social community aspects either. Only 13 of the tested apps provided a connection to a social media network. This requires improvement, as previous findings suggest that users appreciate the opportunity to share data with designated individuals [35]. As some of the apps were trial versions, it is reasonable to assume that these apps might include more features in the paid version; yet this aspect only applied to 5 of the tested apps. The 4 apps that offered the additional use of a wearable device might also offer additional features, which were not covered by this study. Whereas registration and the need of a password for working with the app could make the use of apps with sensible data much more secure, this was not the case for the current sample. Furthermore, a considerable number of the sample included either permanent or pop-up advertisement. Both pose serious security risks, as they often use unsafe mechanisms [69] and should, therefore, be avoided. As this study, however, focused only on free apps, the large number of apps including advertisement might not be representative for paid apps. Regardless of this, the use and effects of aspects such as advertisement and the prerequisite of an Internet connection should be investigated in future studies.

### Limitations

In view of the MDA framework by Hunicke and colleagues [41], this study investigated the use of gamification on the level of gamification techniques. This level is visible to the user and can greatly influence his or her behavior and reactions. Moreover, in opposite to dynamics and aesthetics, this level of gamification techniques can be directly influenced by app designers. Thus, the aim of this study was to find out whether app designers are currently trying to influence the user’s behavior through the implementation of gamification techniques. For this purpose, an expert review of the apps was conducted. However, as no randomized controlled trials were carried out in this study, it is impossible to make any affirmative conclusions about the effects of the investigated techniques. Future studies should, therefore, focus on the levels of dynamics and aesthetics to examine the effects that the use of the gamification techniques investigated in this study might have on the user’s behavior and reactions.

Whereas this study investigated an important aspect with its focus on the quantitative analysis of gamification usage in the sample, it needs to be kept in mind that the integration of game elements alone is no guarantee for successful gamification [33]. Hence, future studies should also concentrate on a qualitative analysis of the gamification techniques used in stress management apps in order to supplement the data gained in this study. On one hand, such studies should focus on the way gamification techniques are implemented. Another area of investigation that should be focused on in future work is the general quality of the investigated apps. This includes aspects such as color, appearance, and design of the app; ease of use; as well as other usability aspects.

Regarding the implications of this study, one must keep in mind that the results of the correlational analysis are hard to interpret due to the low mean and the standard deviation of the number of gamification techniques in the sample. A possible reason for the low mean of gamification techniques in the current sample could be that this review only focused on free apps. This may have resulted in the exclusion of paid apps with a more extensive use of gamification. Nonetheless, according to AppBrain [70], 90% of available android apps in the category “health and fitness” and 86% in the category “medical” are available for free at this point. Thus, this study should be representative to some degree.

### Conclusions

The results of this study clearly reveal that the use of gamification techniques in stress management apps is not very common. This is the case for the implementation of gamification techniques as well as the association of those techniques with evidence-based content (use of behavior change techniques and stress management methods [54]). It, therefore, needs to be concluded that app designers are not trying to influence user behavior through the implementation of gamification at this point. In view of gamification’s positive effect on motivation and engagement [21], app designers should, however, consider making more comprehensive use of gamification techniques in order to increase user compliance. In addition to this, developers should pay intense attention to the context and overall aim of the app when selecting techniques, as not all techniques appear suitable for every context. With this in mind, the cooperation of experts in the fields of gaming, behavior change theory, and stress management seems imperative to ensure a solid combination and effectiveness of techniques. If followed, this strategy has the potential to greatly enhance the effectiveness of apps aimed at stress management and other behavioral changes. Nonetheless, future studies should examine the effects of gamification techniques and their combination with behavior change techniques and stress management methods on the user in randomized controlled studies.

This study was the first to investigate the use of gamification techniques as well as the cooccurrence of gamification techniques and evidence-based content in stress management apps. Moreover, it provides an extended framework for the investigation of gamification usage in mHealth apps.

## Appendix

Multimedia Appendix 1 Gamification score for each reviewed stress management app from the Google Play Store.

## Abbreviations

3-D: three-dimensional
AC: agreement coefficient
e-book: electronic book
