# Supplementary material for: Gamification in Stress Management Apps: A Critical App Review
Source: JMIR Serious Games. 2017 Jun 7;5(2):e13. doi: 10.2196/games.7216 (PMC5480012; doi:10.2196/games.7216)
Supplement: Multimedia Appendix 1 [file games_v5i2e13_app1.pdf]

App name	App provider	Gamification technique score
101 Ways to Be Healthy	Life Time Fitness	0
3 Minute Stress Buster	Astute Software	0
5 Best Health Tips	Eurekafox	0
Anti Stress	reism	0
Anti-Stress-	Aps Studios	0
Anti-stress Exercise	QUO?TE developers	0
Best Guided Meditation	Muhhas	0
Best Self-Help Quotes	Waikiki Sky	0
Best Wisdom Quotes	Waikiki Sky	0
Beste entspannende Musik	Aps Studios	0
Dance Workout	Marinapps	0
Der Klang der regen	Aps Studios	0
Dr Yousef	Mobile App Company	0
EFT Tapping Simulation	Mikhail Game Tech	0
Entspannende Musik	Aps Studios	0
Entspannungsmusik	Aps Studios	0
Essential Oils for Stress	Almasi	0
Exercise - A Necessity	ACIW	0
Free Meditation - Take a Break	Meditation Oasis	0
Get Out of Stress	AndroAppDevelopers	0
Health Foods Now	TopFreeAppsTips	0
Meditation Music	MeTapps	0
Motivational Quotes	Waikiki Sky	0
Motivational Quotes for Stress	Tiger Shark Pendekar	0
Natural Stress Relief Hypnosis	Mastermind App	0
Preksha Meditation*	Preksha International	0
Regen ton	Aps Studios	0
Regen und Donner Geräusche	Aps Studios	0
Reiki Heal	Devils Canon	0
Social BrainGym Lite	Brain Relax Technosoft	0
Stress Flush	ENKI	0
Stress Free Live Free	Davidandroidbro	0

App name	App provider	Gamification technique score
Stress Management	EclipseBoy	0
Stress Reduction Audio	Mindware Consulting, Inc	0
Stress Relief Free Guide	AppBelle	0
Stress Relief Hypnosis	Shoonger	0
stress relief reduce tensions	malcolm pugh	0
Stressheads	YouthNet	0
Sun Anywhere	Deux	0
Tiefe Meditation Musik	Aps Studios	0
Wellen strand klingen	Aps Studios	0
WorkLIFE 365	Lindsey Witmer Collins	0
AnandaYoga	ECHK Hong Kong	1
Breathe2Relax	T2	1
Calm-IT Stress Relief	New Oceans	1
eSense Temperature	Mindfield Biosystems Ltd.	1
INNOVZEN	INNOVZEN	1
O2CHAIR	INNOVZEN	1
POP POP	BIGTEXAPPS	1
Reiki-Energie	Dog Breeds Apps	1
Stress Check	kzk	1
Stress Management	Nasim	1
Stress Zapper	Green Infinity	1
The Stress Surfer	Workforce Management tems	1
Baoding Stress	GaMoon	2
Luftpolsterfolie - Stressabbau	ExaMobile S.A.	2
Mevii	Thrive 4-7	2
Pocket Clarity: Mindfulness	Three Pound	2
RELAXATION OPTIMIZER	IntelaText	2
Stress Check by Azumio	Azumio Inc.	2
Stress Management Guide	DHMobiApp	2
StressLocator Free	Happy Electronics, s.r.o.	2
